# Supplementary material for: A pH-responsive complex based on supramolecular organic framework for drug-resistant breast cancer therapy
Source: Drug Deliv. 2021 Dec 24;29(1):1–9. doi: 10.1080/10717544.2021.2010839 (PMC8725986; doi:10.1080/10717544.2021.2010839)
Supplement: Supplemental Material [file IDRD_A_2010839_SM7594.docx]

**Supplementary material**s

**Figure S1** ^1^H NMR spectra (400 MHz) of the mixtures of TAH (1.0 mM) with CB[8] (2eq.) in D_2_O at 25 °C.

**Figure S2** Synthetic route of TAH and ^1^H NMR spectra (400 MHz) of TAH (1.0 mM) in D_2_O at 25 °C.

**Figure S3** ^1^H NMR spectra (400 MHz) of the SOF (1.0 mM) at different time point in D_2_O at 25 °C.

**Figure S4** The hydrodynamic diameter (D_H_) of SOF (0.2 mM) determined by DLS (day 0 and day 14).
